# The critical immune basis for differential responses to immunotherapy in primary versus metastatic pancreatic cancer

**DOI:** 10.18632/oncotarget.28373

**Published:** 2023-04-24

**Authors:** Brian Diskin, Sarah Schwartz, George Miller

**Keywords:** pancreatic cancer, liver metastasis, immunnotherapy

Emerging clinical trials investigating the potential benefits of immunotherapy in pancreatic ductal adenocarcinoma (PDA) have failed to show a significant improvement in survival [1]. This disappointment belies the fact that PDA has been “cured” or its growth slowed innumerable times in sophisticated pre-clinical PDA mouse models. The clinical resistance of PDA to immunotherapy has been presumptively assigned to the tolerogenic innate and adaptive immune infiltrate in PDA and is also frequently attributed to a characteristically hypoxic and fibrotic tumor microenvironment (TME) that is inaccessible to immune cells, which additionally have tolerogenic features [2]. PDA most commonly metastasizes to the liver. The liver harbors a rich diversity of innate immune populations including NK cells, Kupffer cells, NKT cells, double negative T cells [3, 4]. Nevertheless, the liver is a uniquely immune-tolerant organ. The most conspicuous example of this in oncology is the liver’s status as the most common location for metastasis from gastrointestinal cancers [5]. In fact, the liver is a site of metastases in approximately 90% of patients with advanced PDA. The potentially divergent responses to immunotherapy in the respective environments of primary versus metastatic PDA within the same host has not been well-studied. It is an unfortunate fact that all failed clinical trials assessing immunotherapeutic efficacy were conducted in metastatic PDA, whereas basic pre-clinical investigations are usually performed in primary PDA using genetically engineered mouse models. We postulated that this dichotomy may explain the gap between preclinical promise and ultimate clinical failure. We discovered that the respective TMEs of primary PDA and liver metastases differ markedly and this fact plays a critical role in dictating site-specific PDA response to immunotherapy [6].

In our recent publication, we show that PDA liver metastases are uniquely resistant to T cell based immunotherapies – in sharp contrast to the immunotherapeutic responsiveness of primary PDA [6]. The basis for this observation is the their starkly different respective immune TMEs. In interrogating the metastatic immune compartment in the liver, we demonstrated that the TME is infiltrated by highly anergic T cells and MHCII^lo^IL10^+^ M2-like macrophages that are unable to present tumor-antigen. The underlying driver of this innate and adaptive immune tolerance in PDA liver metastases was metastases-infiltrating B cells. We found that B cells constituted ~25% of the tumor-infiltrating lymphocytes in metastatic PDA liver deposits compared to ~10% in primary PDA. Further, we discovered a novel population of CD24^+^CD44^–^CD40^–^ B cells in the metastatic liver, which is recruited to the metastatic milieu by *Muc1^hi^IL18^hi^* tumor cells. This distinct sub-population of tumor cells is enriched >10-fold in liver metastases compared to primary PDA. We further discovered that the predominant population of liver metastases-infiltrating B cells in-turn trigger macrophage-mediated adaptive immune-tolerance via CD200 and BTLA. Conversely, by targeting B cells or blocking CD200/BTLA, we demonstrated enhanced macrophage and T-cell immunogenicity, which enabled immunotherapeutic efficacy of liver metastases. Unlike liver metastases, the primary PDA tumor sites lack this distinctive *Muc1^hi^IL18^hi^* tumor cell-driven B cell infiltrate and are thus characterized by a more immunogenic macrophage and effector T cell population. Hence, the immunotherapeutic responsiveness of primary PDA is far more robust. Besides showing a targetable pathway for immunotherapeutic response in metastatic liver PDA and highlighting the divergent immune landscapes and mechanistic underpinnings for distinct responsiveness to immunotherapy in each compartment, our data suggest that models of primary PDA are poor surrogates for evaluating immunity or treatment response in advanced disease.
